# Prognostic value of the post-exercise heart rate recovery and BHDE-index in chronic obstructive pulmonary disease

**DOI:** 10.1186/s12890-023-02557-7

**Published:** 2023-07-17

**Authors:** Shih-Yu Chen, Chun-Kai Huang, Chia-Ling Wu, Hui-Chuan Peng, Chong-Jen Yu, Jung-Yien Chien

**Affiliations:** 1 Department of Internal Medicine, National Taiwan University Hospital Hsin-Chu Branch, Hsinchu City, Taiwan; 2 Graduate Institute of Clinical Medicine, National Taiwan University College of Medicine, Taipei, Taiwan; 3 Department of Internal Medicine, National Taiwan University Hospital, National Taiwan University College of Medicine, Taipei, Taiwan; 4 Institute of Epidemiology and Preventive Medicine, College of Public Health, National Taiwan University, Taipei, Taiwan; 5 Department of Integrated Diagnostic & Therapeutics, National Taiwan University Hospital, Taipei, Taiwan; 6 Department of Nursing, National Taiwan University Hospital, National Taiwan University College of Medicine, Taipei, Taiwan

**Keywords:** Pulmonary rehabilitation, Chronic obstructive pulmonary disease, Pulmonary function, Heart rate recovery

## Abstract

**Background:**

The BODE index, consisting of body mass index (B), airflow obstruction (O), dyspnea score (D), and exercise capacity (E), can predict outcomes in COPD. However, when spirometry was restricted to prevent cross-infection such as COVID-19 pandemic, a modified index would be needed. Because cardiovascular dysfunction is associated with poor clinical outcomes in COPD, we conducted a novel BHDE-index by replacing spirometry with post-exercise heart rate recovery (HRR, H) and evaluated its predictive performance in this observational study.

**Methods:**

From January 2019 to December 2019, enrolled patients were analyzed as a derivation cohort for the setup of the model. This model was verified in another group of patients generated between January 2020 and December 2020, as the validation cohort. The post exercise HRR was defined as the difference of heart rate immediately after and 1 min after test cessation.

**Results:**

A total of 447 patients with COPD were enrolled. Patients with abnormal HRR were older, with more severe airway obstruction, severe airway symptoms, faster resting heart rate, shorter 6-min walk distance and higher frequency of severe acute exacerbation in previous one year. The prediction performance of the BHDE-index for one-year severe COPD exacerbation was similar to that of the BODE-index in both the derivation and validation groups [area under the receiver operating characteristic curve (AUROC) 0.76 vs. 0.75, p = 0.369; AUROC 0.74 vs. 0.79, p = 0.05]. The prediction performance for 1 year mortality was also similar between BHDE-index and BODE-index in both cohorts [AUROC 0.80 vs. 0.77, p = 0.564; 0.76 vs. 0.70, p = 0.234]. Univariate and multivariate analyses also showed that the BHDE-index was an independent and important predictor of annual severe COPD exacerbation in the derivation and validation cohorts.

**Conclusions:**

The BHDE-index is a good and easy-to-perform prediction model for the risk of severe acute exacerbation and 1-year mortality in COPD wherever spirometry results are unavailable.

**Supplementary Information:**

The online version contains supplementary material available at 10.1186/s12890-023-02557-7.

## Introduction

Chronic obstructive pulmonary disease (COPD), a non-communicable lower respiratory tract illness, is the third leading cause of death worldwide [1]. Without proper control, patients with COPD may suffer from frequent acute exacerbation, have poor quality of life, and bear a huge socioeconomic burden [2, 3]. Predicting clinical outcomes is critical in the follow-up of patients with COPD, and many efforts have been devoted to developing prediction models. Currently, there are no single clinical parameters or biomarkers that fit one-for-all for such heterogeneous complex disease [4–6]. The body mass index, airflow obstruction, dyspnea, and exercise capacity (BODE) index proposed by Celli et al. in 2004 has been proven to be a good prediction model of mortality in patients with COPD [7].

However, in the era of the COVID-19 pandemic, airflow obstruction evaluation using spirometry, a potential aerosol-generating examination, cannot be widely performed. Due to safety concerns, strict guidance has been applied to the spirometry procedure to protect staff and prevent patients from cross-infection [8]. A substitute parameter that is easy to perform with less risk of aerosol generation is needed.

Cardiovascular disease is the major comorbidity of COPD and is closely associated with clinical outcomes [9, 10]. Post-exercise heart rate recovery (HRR) has been proposed as an important and practical predictive factor of cardiovascular outcome [11–13]. The prognostic value of heart rate and its degree of attenuation after exercise has also been illustrated in several studies among patients with COPD [14–16]. Hence, the addition of post-exercise HRR to a multidimensional model may be reasonable and may enhance predictability. In this study, we hypothesized that the incorporation of the parameter of post-exercise HRR would generate a new modified multidimensional model with the same capability as the BODE model.

## Study Design and methods

### Study design

This observational study was conducted at the National Taiwan University Hospital, a university-affiliated medical center in Taiwan. The study involved two groups of patients: a derivation cohort for the build-up of the modified index, and a validation cohort to test its feasibility. Between January 2019 and December 2019, patients were enrolled as the derivation cohort, and a validation cohort was generated from patients enrolled between January 2020 and December 2020. All the participants were spirometry-confirmed COPD patients who were enrolled in the Taiwan nationwide COPD pay-for-performance program [17]. In brief, the pay-for-performance program was launched in April 2017, and patients enrolled in this program received comprehensive pharmacologic and non-pharmacologic therapies based on the Taiwan’s COPD guidelines. The patients were recommended to maintain regular follow-up every three months, and patients’ evaluations, prescriptions, and episodes of acute exacerbation were regularly assessed and recorded. Patients who had past history of lung surgery, atrial fibrillation, heart failure, pacemaker placement, currently under rate or rhythm control medication or severe neurological deficits that prevent patients from following simple orders or completing whole course of 6-minute walking test were excluded. All participants received thorough pulmonary rehabilitation evaluation at the time of enrollment.

The diagnostic criteria for COPD are based on spirometry findings of post-bronchodilator forced expiratory volume in one second (FEV1) to forced vital capacity (FVC) ratio (FEV1/FVC ratio) of less than 70%, a consensus of the Global Initiative for Chronic Obstructive Lung Disease (GOLD) Report [18]. The 6-min walking test was performed at COPD diagnosis, according to the guidelines of the American Thoracic Society Committee on Proficiency Standards for Clinical Pulmonary Function Laboratories [19]. Heart rate was measured by a pulse oximeter during the walk test. The HRR was calculated as the difference between the heart rate at the end of test and heart rate after 1 min of the test cessation with patients at standing position. In the derivation cohort, a receiver operating characteristic (ROC) curve of HRR, predicting the incidence of severe acute exacerbation was constructed. A cut-off point of 11 beats per minute was selected by calculating the Youden’s index from the coordinates of the curve. An abnormal post-exercise HRR was defined as a heart rate difference of less than 11 beats per minute (supplementary figure S1). Another ROC curve of HRR, predicting the incidence of severe acute exacerbation was made in the validation cohort, and by calculating the Youden’s index from the coordinates of the curve, 11 beats per minute was still the optimal cut-off for categorizing the status of HRR (supplementary figure S1).

### Data collection and follow-up

The patients’ baseline characteristics, spirometry results, smoking status, severity of symptoms, and pulmonary and cardiology prescriptions were documented. Additionally, the parameters of the 6-min walking test and the heart rate at baseline, peak, end-test, and 1-min post-test were all recorded. According to the GOLD report, a severe acute exacerbation of COPD was defined as a disease status that necessitates emergency department visits or hospitalization. The details of the occurrence of severe exacerbations were gathered from medical records. The time interval between the first day of pulmonary rehabilitation evaluation and the first episode of acute severe exacerbation was analyzed. This study was approved by the institutional review board of the National Taiwan University Hospital (201905058RINB).

### Statistical analysis

Continuous parameters were compared using the Wilcoxon rank-sum test. Differences in categorical parameters were analyzed using the chi-squared test or the Fisher’s exact test, as appropriate. The variables are presented as numbers (percentages), median (range), or mean ± standard deviation (SD), as appropriate. A two-sided p-value of 0.05 is considered statistically significant in the present study. The correlation between parameters were examined using Spearman’s rank correlation. The ROC and area under the ROC (AUROC) curves were used to compare the predictivity of different indices. The Cox proportional hazard model and log-rank test were used to analyze the hazard ratios (HRs) of different predicting factors, pertaining the incidence of acute severe exacerbation. All statistical analyses were performed using STATA version 14 software (StataCorp LLC, TX, USA).

## Results

### Baseline demographics

**Fig. 1:**
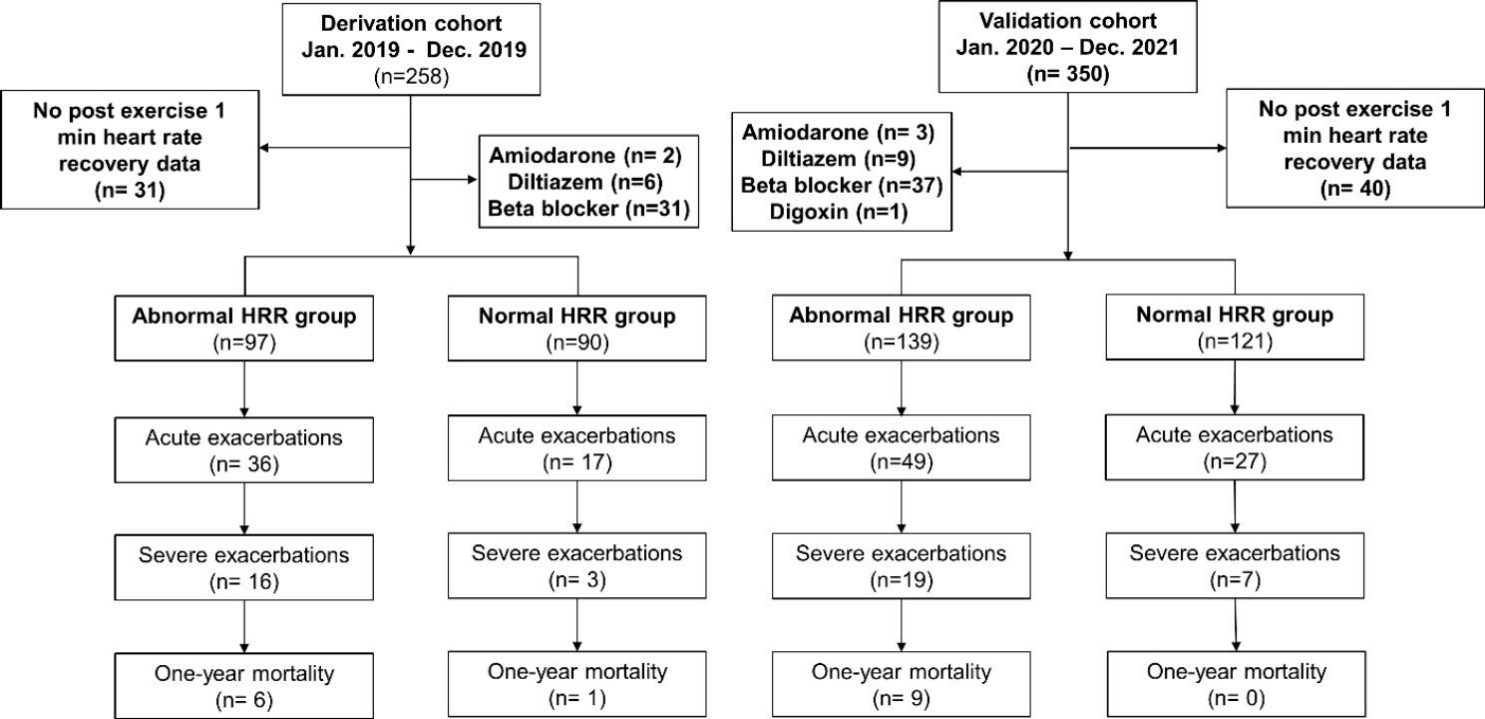
Flow chart of the enrollment. HRR, post-exercise heart rate recovery

A total of 187 and 260 patients with spirometry-diagnosed COPD completed the 6-min walking test, and thorough evaluation was performed in the derivation and validation cohorts, respectively (Fig. 1). The baseline characteristics of age, sex, weight, height, body mass index, spirometry data, COPD Assessment Test (CAT), smoking status, resting heart rate, heart rate recovery and 6-min walking test results are shown in Table 1. Significantly fewer active smokers were found in the validation cohort than in the derivation cohort.

**Table 1:** Baseline characteristics of all participants

	Total	Derivation cohort	Validation cohort	p value
Number of patients	447	187	260	
Age, years	71.8 (9.5)	71.3 (9.6)	72.1 (9.4)	0.350
Male	402 (89.9)	172 (92.0)	230 (88.5)	0.223
Weight, kg	63.3(11.2)	63.5 (11.3)	63.1 (11.2)	0.757
Height, cm	163.9 (6.4)	164.4 (5.9)	163.7 (6.7)	0.213
Body mass index, kg/m^2^	23.5 (4.0)	23.5 (4.2)	23.6 (3.9)	0.840
FEV1, % predicted	65.8 (25.5)	64.6 (24.2)	66.6 (26.5)	0.418
CAT score	6 (0,30)	6 (0,28)	7 (0,30)	0.976
Current smoker	70 (15.7)	40 (21.4)	30 (11.5)	0.005
Resting heart rate, bpm	82 (47,139)	82 (48,139)	81 (47,131)	0.289
6-min walk distance, m	372.9 (117.3)	378.6 (115.6)	368.8 (118.5)	0.387
6-min walk distance, % of predicted value	80.3 (23.0)	80.9 (22.5)	79.8 (23.3)	0.592

Data are presented as n (%), mean (standard deviation), or median (minimum or maximum)

FEV1, forced expiratory volume in 1 s; CAT, chronic obstructive pulmonary disease assessment test

### Demographics influenced by HRR status

After combining the patients of the derivation and validation cohorts, 236 participants tested positive for the post-exercise HRR and were designated as the abnormal recovery group. The remaining participants were included in the normal recovery group. Table 2 shows that patients in the abnormal recovery group were elder (73.4 vs. 69.9 years, p < 0.05), more male patients (93% vs. 87%, p < 0.05), had worse FEV1 performance (61.0% vs. 71.1%, p < 0.05), higher resting heart rate (85 vs. 78 beats/min, p < 0.05), worse 6-min walking distance performance (331.0 vs. 419.8 m, p < 0.05) and more patients with at least one episodes of severe acute exacerbation in previous one year (12.3% vs. 4.7%, p < 0.05) than the normal recovery group.

**Table 2:** Comparison of baseline characteristics among participants with normal or abnormal heart rate recovery in a combination of derivation and validation cohorts

	Post 6-min-walk test HRR		
	Normal (HRR ≥ 11 bpm)	Abnormal (HRR < 11 bpm)	p value
Number of patients	211	236	
Age, years	69.9 (9.9)	73.4 (8.8)	< 0.05
Male	183 (86.7)	219 (92.8)	0.033
Weight, kg	63.5 (10.3)	63.1 (12.0)	0.678
Height, cm	164.0 (6.5)	163.9 (6.3)	0.863
Body mass index, kg/m^2^	23.6 (3.6)	23.5 (4.3)	0.728
FEV1, % predicted	71.1 (23.4)	61.0 (26.5)	< 0.05
CAT score	5 (0,27)	8 (0,30)	< 0.05
Current smoker	32 (15.2)	38 (16.1)	0.074
Resting heart rate, bpm	78 (47,131)	85 (48,139)	< 0.05
Heart rate recovery, bpm	17 (11,55)	5 (0,10)	< 0.05
6-min walk distance, m	419.8 (93.7)	331.0 (120.4)	< 0.05
≥ 1 severe AE in previous 1 year	10 (4.7)	29 (12.3)	< 0.05

Data are presented as n (%), mean (standard deviation), or median (minimum, maximum)

CAT, COPD assessment test; FEV1, forced expiratory volume in 1 s; HRR, heart rate recovery; AE: acute exacerbation

Patients with abnormal HRR had a higher incidence rate of severe acute exacerbation than those with normal HRR (incidence rate ratio 7.41, p < 0.05). The Kaplan-Meier time-to-event analysis also showed a higher incidence of severe acute exacerbation in patients with abnormal HRR than in those with normal HRR in both cohorts [HR, 5.39; 95% confidence interval (CI),1.57–18.51, p < 0.05; supplementary figure S2A and HR, 2.43; CI, 1.02–5.77, p < 0.05; supplementary figure S2B]. The correlation coefficients between HRR and CAT, breathlessness score (mMRC, resting Borg score, post-exercise Borg Score) and 6-minute walking distance were − 0.115, -0.274, -0.109, -0.067, 0.252 in derivation cohort and − 0.239, -0.386, -0.166, -0.103, 0.397 in validation cohort, respectively (supplementary figure S3).

### BODE index versus modified multidimensional index in the prediction of severe acute exacerbation of COPD

**Fig. 2:**
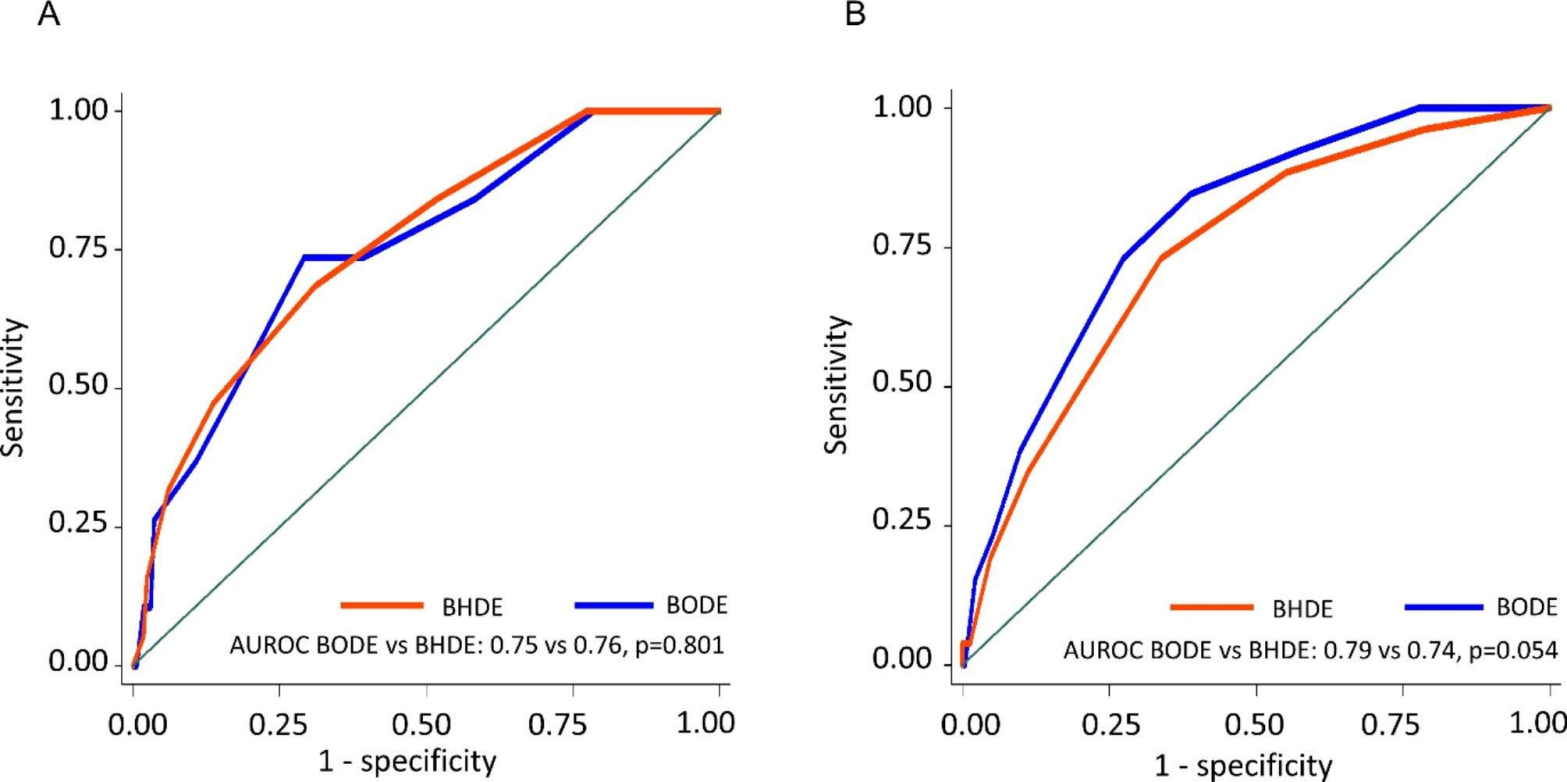
Receiver operating characteristic curves comparing BODE with BHDE in the derivation cohort (2**A**), and validation cohort (2**B**) in the prediction of occurrence of severe acute exacerbation. B, body mass index; O, airflow obstruction; H, post 6-minute walk test 1-min heart rate recovery; D, dyspnea score; E, exercise intolerance; AUROC, area under the receiver operating characteristic curve

We further incorporated HRR (H) to replace spirometry in the BODE-index to form a new multidimensional index, namely the BHDE-index, where abnormal HRR is given a score of 1 and a score of 0 for normal ones (Supplementary Figure S4). BHDE-index tend to have better predictive performance than HRR alone for severe acute exacerbation in both derivation cohort and validation cohort (AUROC 0.76 vs. 0.72, p = 0.572; 0.74 vs. 0.66, p = 0.085, supplementary Figure S5). BHDE-index had slightly better predictive power for severe acute exacerbation than BDE-index in derivation cohort but were equal in validation cohorts (AUROC 0.76 vs. 0.73, p = 0.169, 0.74 vs. 0.74, p = 0.899, supplementary Figure S6). The prediction performance of BHDE was comparable with that of the BODE index in the derivation cohort (AUROC 0.76 vs. 0.75, p = 0.801; Fig. 2A) and remained similar to that of BODE in the validation cohort (AUROC, 0.74 vs. 0.79, p = 0.054; Fig. 2B).

**Fig. 3:**
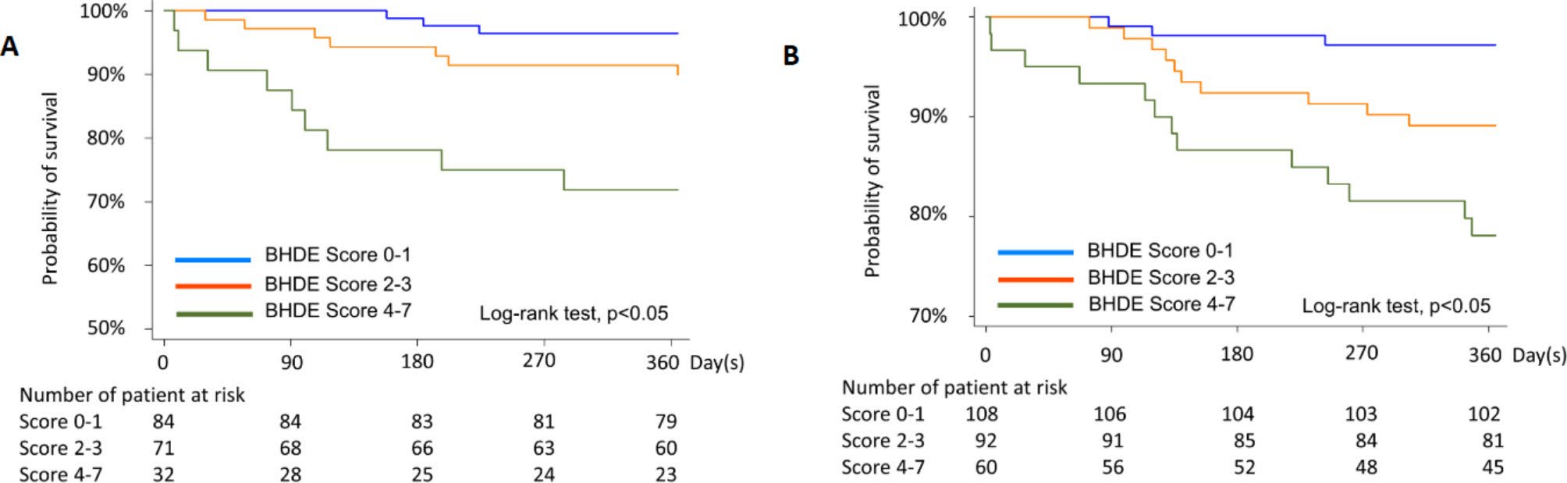
Kaplan-Meier time-to-event plot and log-rank test for time to first acute severe exacerbation in one year, according to the score of BHDE in the derivation (**3A**) and validation (**3B**) cohorts. B, body mass index; O, airflow obstruction; H, post 6-minute walk test 1-min heart rate recovery; D, dyspnea score; E, exercise intolerance.

The HR for acute severe exacerbation with each one-point increment in BHDE is 1.59 (95% CI, 1.23–1.98) in the derivation cohort. The patients were stratified into three groups, according to their total scores: scored 0–1 vs. scored 2–3 vs. scored 4–7. The Kaplan-Meier survival analysis with BHDE in accordance with the three groups showed that patients with a lower total score were associated with a reduced risk of acute severe exacerbation in the derivation (Fig. 3A) and validation cohorts (Fig. 3B). Univariate and multivariate analyses of factors associated with acute severe exacerbation of COPD revealed that BHDE was an independent and important risk factor in the derivation and validation cohorts (Table 3 and supplementary table S1).

**Table 3:** Univariate and multivariate analyses with factors associated with severe acute exacerbation of COPD within one year in the derivation cohort

	Univariable analysis					Multivariable analysis		
	HR	95% CI		p-value		HR	95% CI	p-value
BHDE index	1.59	1.23–1.98		< 0.05		1.45	1.12–1.85	< 0.05
Age, year	1.03	0.98–1.08		0.270		-	-	-
Airway obstruction, FEV1 (%)	0.97	0.95–0.99		< 0.05		0.98	0.96–1.01	0.130
Inhaled corticosteroid	3.80	1.37–10.56		< 0.05		1.81	0.60–5.49	0.293
Active smoking vs. quitted	1.34	0.48–3.73		0.58		-	-	-
Severe AE in preceding 1 year	1.24	1.05–1.46		< 0.05		1.20	0.99–1.45	0.06

HR, hazard ratio; CI, confidence interval; BHDE, body mass index (B), post-exercise heart rate recovery (H), dyspnea score (D), and exercise intolerance (E); FEV1, forced expiratory volume in 1 s; AE: acute exacerbation

### BODE index versus modified multidimensional index in the prediction of one year mortality of COPD

**Fig. 4:**
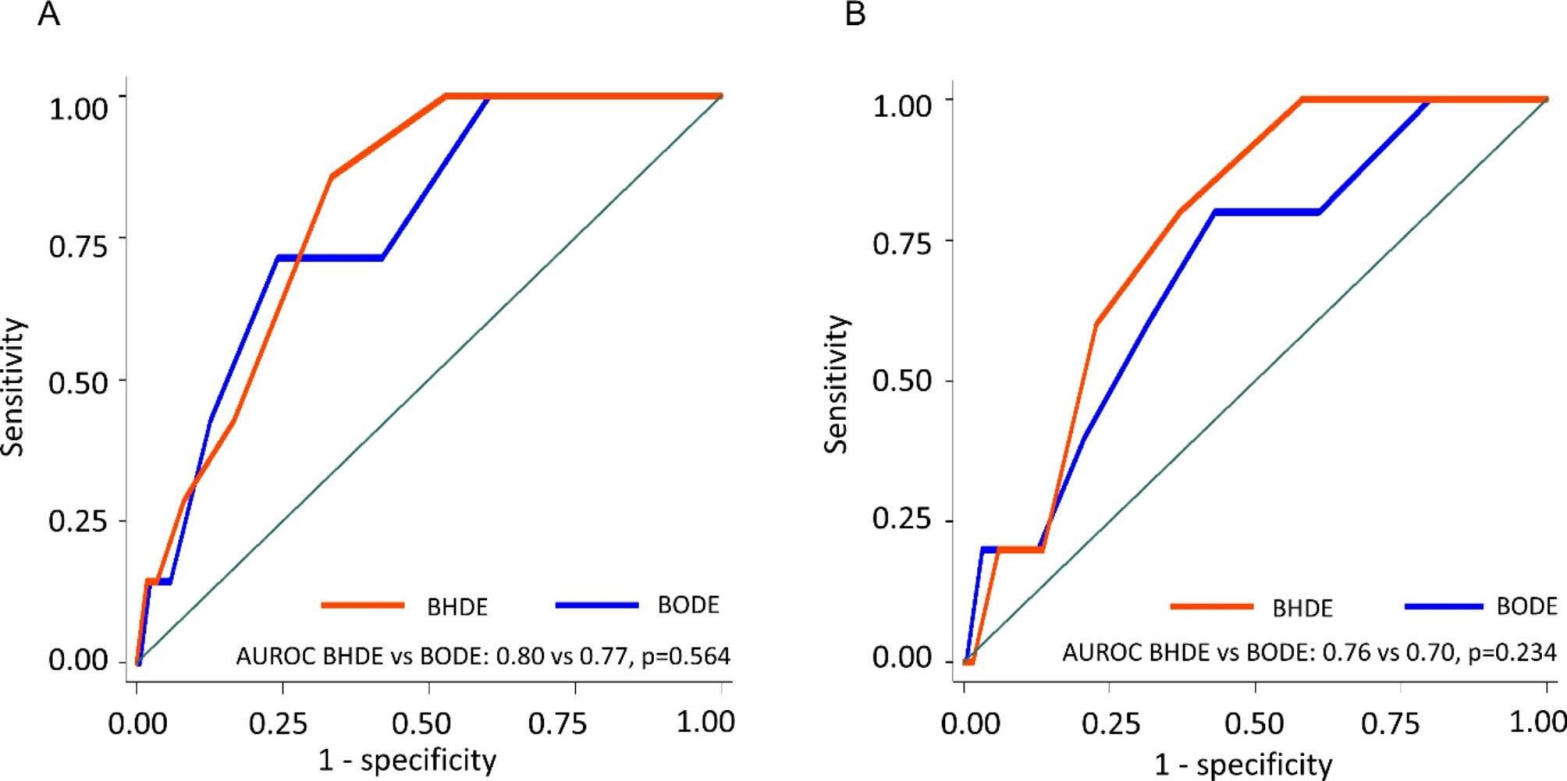
Receiver operating characteristic curves comparing BODE with BHDE in the derivation cohort (4**A**), and validation cohort (4**B**) in the prediction of occurrence of 1-year mortality. B, body mass index; O, airflow obstruction; H, post 6-minute walk test 1-min heart rate recovery; D, dyspnea score; E, exercise intolerance; AUROC, area under the receiver operating characteristic curve

The prediction performance of BHDE-index for one year mortality is better than that of HRR alone in derivation cohort (AUROC 0.82 vs. 0.67, p = 0.006, supplementary Figure S7) and also better than BDE-index in validation cohort (AUROC 0.76 vs. 0.71, p = 0.018 supplementary Figure S8). Figure 4 showed that the prediction performance for one year mortality was comparable between BHDE-index and BODE-index in both cohorts, AUROC 0.80 vs. 0.77, p = 0.564; 0.76 vs. 0.70, p = 0.234 (Fig. 4).

The HR for 1-year mortality with each one-point increment in BHDE is 1.63 (95% CI, 1.14–2.33, p = 0.007) and 1.49 (95% CI, 1.02–2.17, p = 0.04) in the derivation cohort and validation cohort, respectively. Univariate analyses of factors associated with 1-year mortality in derivation cohort showed that BHDE-index, age and severe exacerbation in previous one year were important risk factors. Further multivariate analyses showed that age and severe exacerbation in previous one year remained to be independent risk factors. BHDE-index, although not statistically significant in multivariate analyses, were tend to be associated with increased risk of mortality in both derivation and validation cohorts. (Supplementary table S2 and supplementary table S3).

## Discussion

In the era of the COVID-19 pandemic where spirometry may be limited, the incorporation of a post 6-min walk test 1-min HRR to form a new multidimensional index is a useful alternative in the evaluation of clinical outcomes of patients with COPD.

Except for diagnostic value, there is a growing body of evidence that FEV1 is an unreliable marker pertaining the severity of symptoms and health status impairment [18]. Even the velocity of FEV1 decline was not associated with cardiovascular disease or mortality in patients with COPD [20]. However, in the validation group of this study, we still found that the severity of airflow obstruction was an independent predictive factor for one year risk of severe exacerbation. This may indicate that the decrease in FEV1 with consequent dynamic hyperinflation is still an important part of the pathogenesis of acute exacerbation. Nevertheless, this is only a puzzle regarding the pathogenesis of acute exacerbations. Other factors, such as hypoxia and systemic inflammation, also contribute to both pulmonary and cardiovascular complications through dysregulated heart-lung interactions. An indicator that can better represent the status of heart-lung interaction would be a great substitute for FEV1 to form a new multidimensional model.

In this study, we choose post 6-min walk 1-min HRR as an outcome predictor in the new multidimensional model for several reasons. First, we found that the post 6-min walk 1-min HRR was positively correlated with FEV1. It meets the findings of Seshadri et al. that, in spite of smoking status, the smaller the FEV1, the lower the HRR and the higher the prevalence of abnormal HRR [21]. In the study conducted by *Zhao et al.* abnormal heart rate recovery was associated with worse FEV1% of predicted value, decreased Pi_10_ (a 10-mm airway’s square root of wall area) and even greater airway wall thickness. This association is specifically a feature of patients with COPD because the author found no consistent association between HRR quintiles with spirometry results in normal lung function group or even patients with preserved ratio impaired spirometry [16]. Secondly, a decreased post-exercise HRR in patients with COPD represents autonomic dysfunction, which is the consequence of abnormal lung-heart interaction [15, 22]. It is also well documented to be closely correlated with adverse cardiovascular outcomes. Third, the more severe the dyspnea, the earlier the exercise may be stopped before reaching the maximum heart rate. This leads to a decreased heart rate change during exercise in patient with lung illness. Finally, resting heart rate had been documented to increase with the disease severity of COPD [23, 24]. In other words, the change between peak heart rate would decrease because the baseline heart rate rises. Taken together, post-exercise HRR is a better predictive factor than FEV1 [11, 12]. Furthermore, Lacasse et al. reported that abnormal HRR was a good predictor for mortality in patients with COPD [15]. In this study, we found that patients with abnormal heart rate recovery had a higher incidence rate of severe acute exacerbation than patients with normal heart rate recovery. It correlated with the findings of Rodriguez et al. that the post 6-min walk 1-min HRR is good for acute exacerbation prediction with AUROC being 0.71 (95% CI: 0.60–0.80, p = 0.0001) [14]. The HRR is a potential substitute in multidimensional index where lung function was not available.

In this study, the modified multidimensional index, BHDE, yielded similar predictability for severe acute exacerbation as BODE in the derivation and validation cohorts. Additionally, it is a good risk stratification with a score of 0 to 1 being the best clinical outcome, followed by a score of 2 to 3, and a score of 4 to 7. The modified multidimensional index is easy to perform, and all data can be readily collected in only one pulmonary rehabilitation. It is associated with less risk of biohazard from the spread of respiratory tract infectious disease and provides a useful tool for continued clinical evaluation.

In this study, we selected a cut-off value of 11 beats per minute according to the receiver operation curve of post 6-min walk test 1-min HRR to the possibility of severe acute exacerbation in both derivation cohort and validation cohort. Two previous studies proposed a cut-off value of 14 beats per minute for post 6-minute walk test as a predictor of exacerbation or mortality [14, 15]. But this cut-off point may not be convincing because the sample size of the studies was small. In the study of *Zhao et al.* which analyzed a total of 385 patients from a large data base called COPDGene, a heart rate recovery of 11 beats per minute statistically stratified patients’ outcome in view of 6-minute walk test performance, spirometry results, computed tomography variables and risk of acute exacerbation. Thus, we consider this post-6-minute walk 1-min heart rate recovery threshold practical and suitable in current study.

This study had certain limitations. First, this is a retrospective study and may contain some bias factors, such as loss of acute exacerbation records. However, because all the data were prospectively recorded in the pay-for-performance program and the medical history in other hospitals can be looked up in the e-cloud of the national health insurance system in Taiwan, we are confident of data accuracy. Second, the total follow-up period was only one year. Additionally, a nature selection bias occurred, as patients with illness too severe to be able to perform a 6-min walking test were excluded from this study. Finally, the majority of patients with COPD are male participants, similar to the epidemiology of COPD patients in Taiwan [25]. The results may not be generalizable worldwide.

## Conclusions

In conclusion, post 6-min walk HRR measurement can be a potential outcome predictor for severe acute exacerbation and mortality of COPD. The modified multidimensional index, BHDE, has the same prediction capacity as the BODE index, which is easy to accomplish and has less concern for aerosol biohazards. It is suggested to be used as a clinical evaluation tool when spirometry is unavailable.

## Electronic supplementary material

Below is the link to the electronic supplementary material.

Supplementary Material 1

Supplementary Material 2

Supplementary Material 3

Supplementary Material 4

Supplementary Material 5

Supplementary Material 6

Supplementary Material 7

Supplementary Material 8

Supplementary Material 9

Supplementary Material 10

## Data Availability

All data generated or analyzed during this study are included in this published article and its supplementary information files.

## Abbreviations

AUROC: Area under the receiver operating characteristic curve
BODE index: Index of body mass index, airflow obstruction, dyspnea, and exercise capacity
CAT: COPD Assessment Test
CI: Confidence interval
COPD: Chronic obstructive pulmonary disease
FEV1: Forced expiratory volume in the first second
HRR: Heart rate recovery
HRs: Hazard ratios
Pi_10_: A 10-mm airway’s square root of wall area
ROC: Receiver operating characteristic curve
