# Supplementary figures and images for: Prognostic value of the post-exercise heart rate recovery and BHDE-index in chronic obstructive pulmonary disease
Source: BMC Pulm Med. 2023 Jul 17;23:263. doi: 10.1186/s12890-023-02557-7 (PMC10353238; doi:10.1186/s12890-023-02557-7)

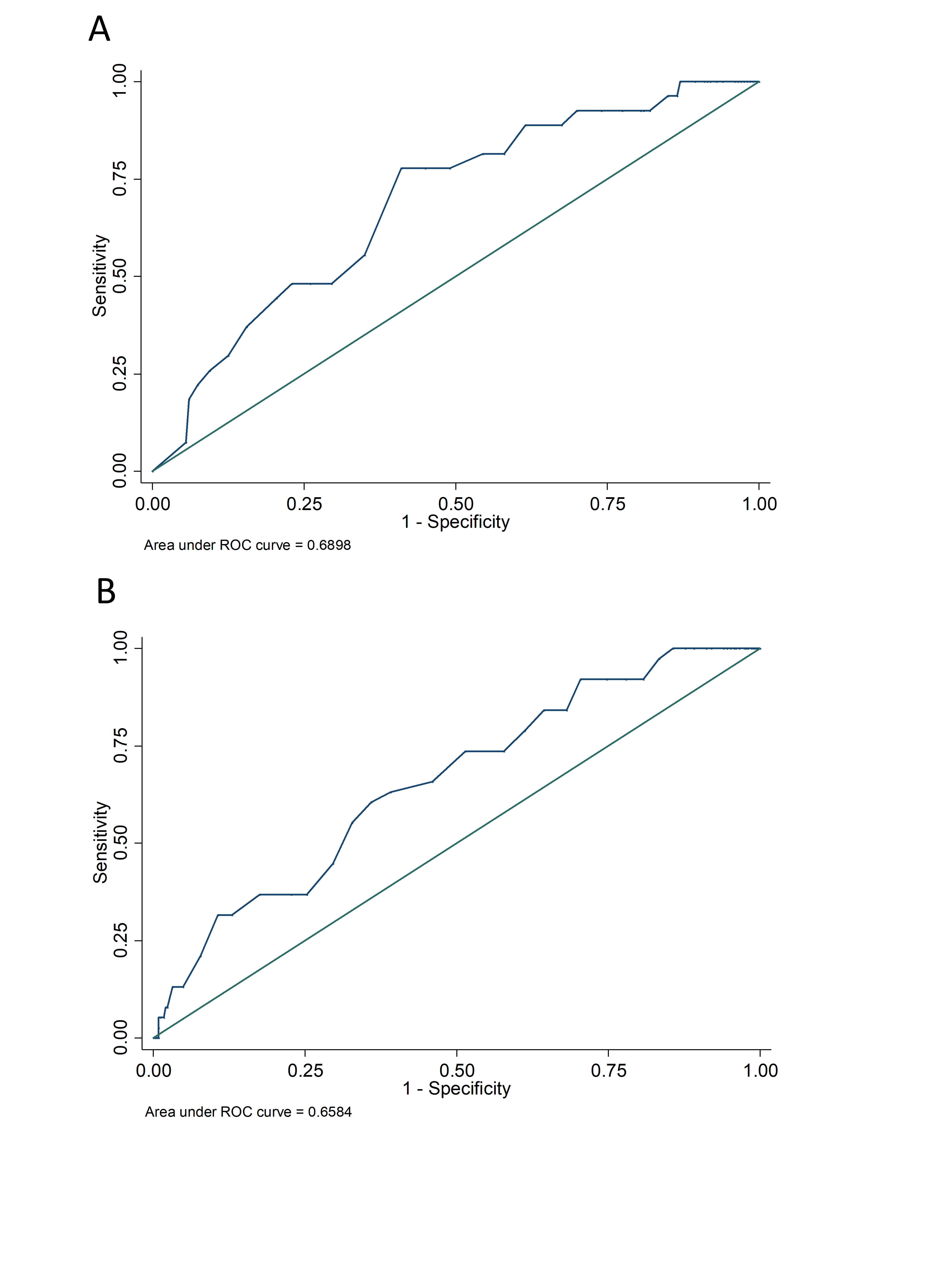

Supplement: Supplementary file 1 — Supplementary Material 1 [file 12890_2023_2557_MOESM1_ESM.tif]

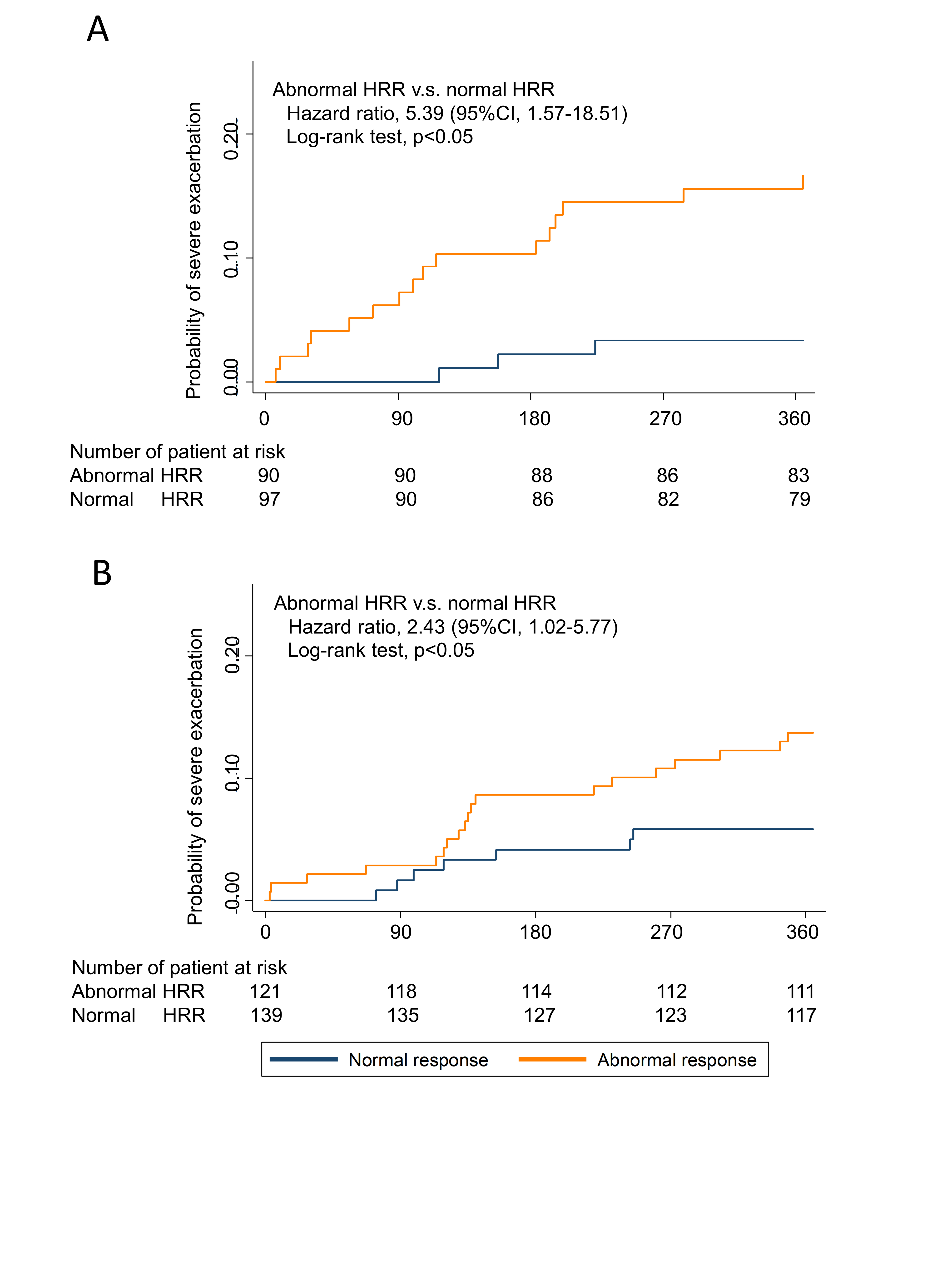

Supplement: Supplementary file 2 — Supplementary Material 2 [file 12890_2023_2557_MOESM2_ESM.tif]

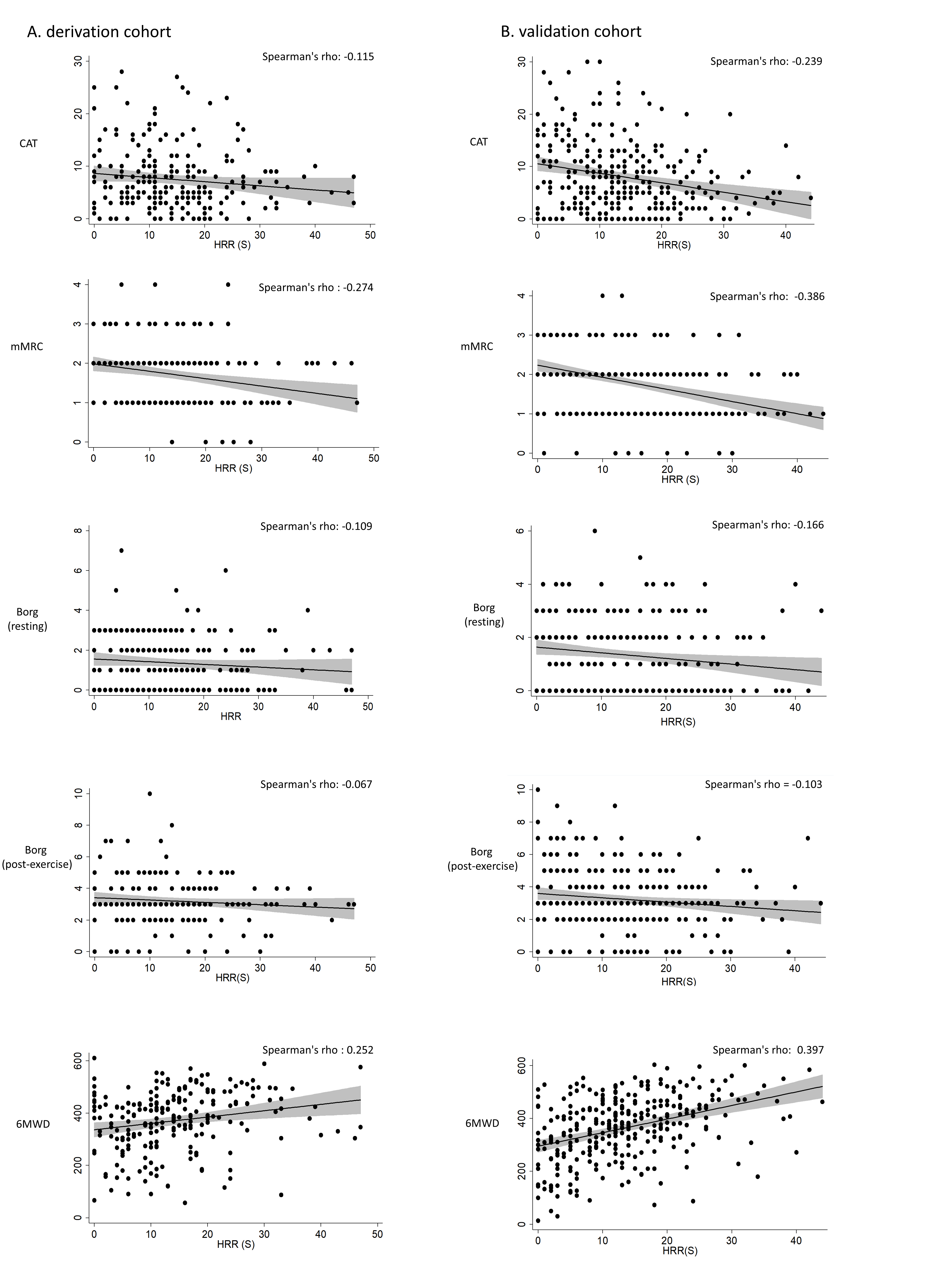

Supplement: Supplementary file 3 — Supplementary Material 3 [file 12890_2023_2557_MOESM3_ESM.tif]

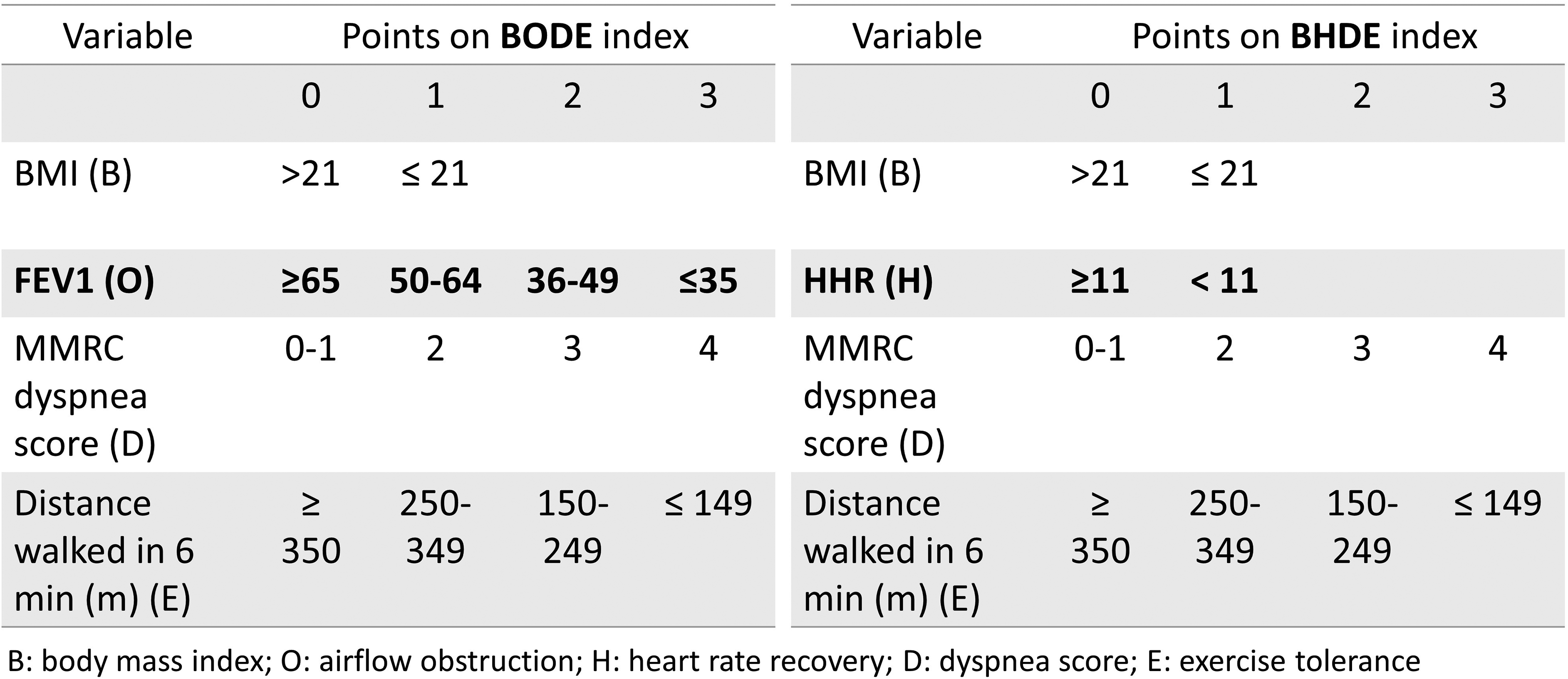

Supplement: Supplementary file 4 — Supplementary Material 4 [file 12890_2023_2557_MOESM4_ESM.tif]

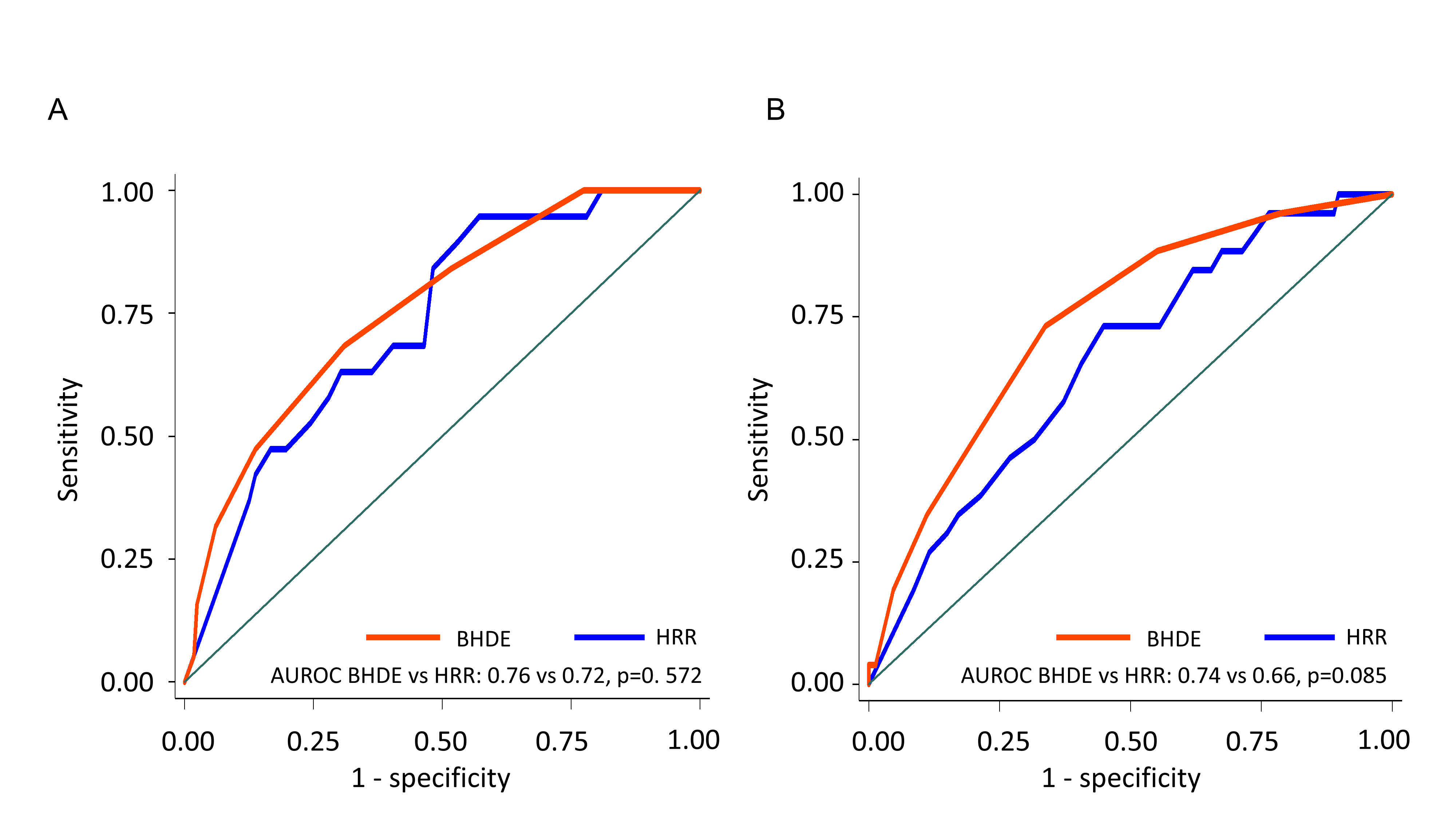

Supplement: Supplementary file 5 — Supplementary Material 5 [file 12890_2023_2557_MOESM5_ESM.tif]

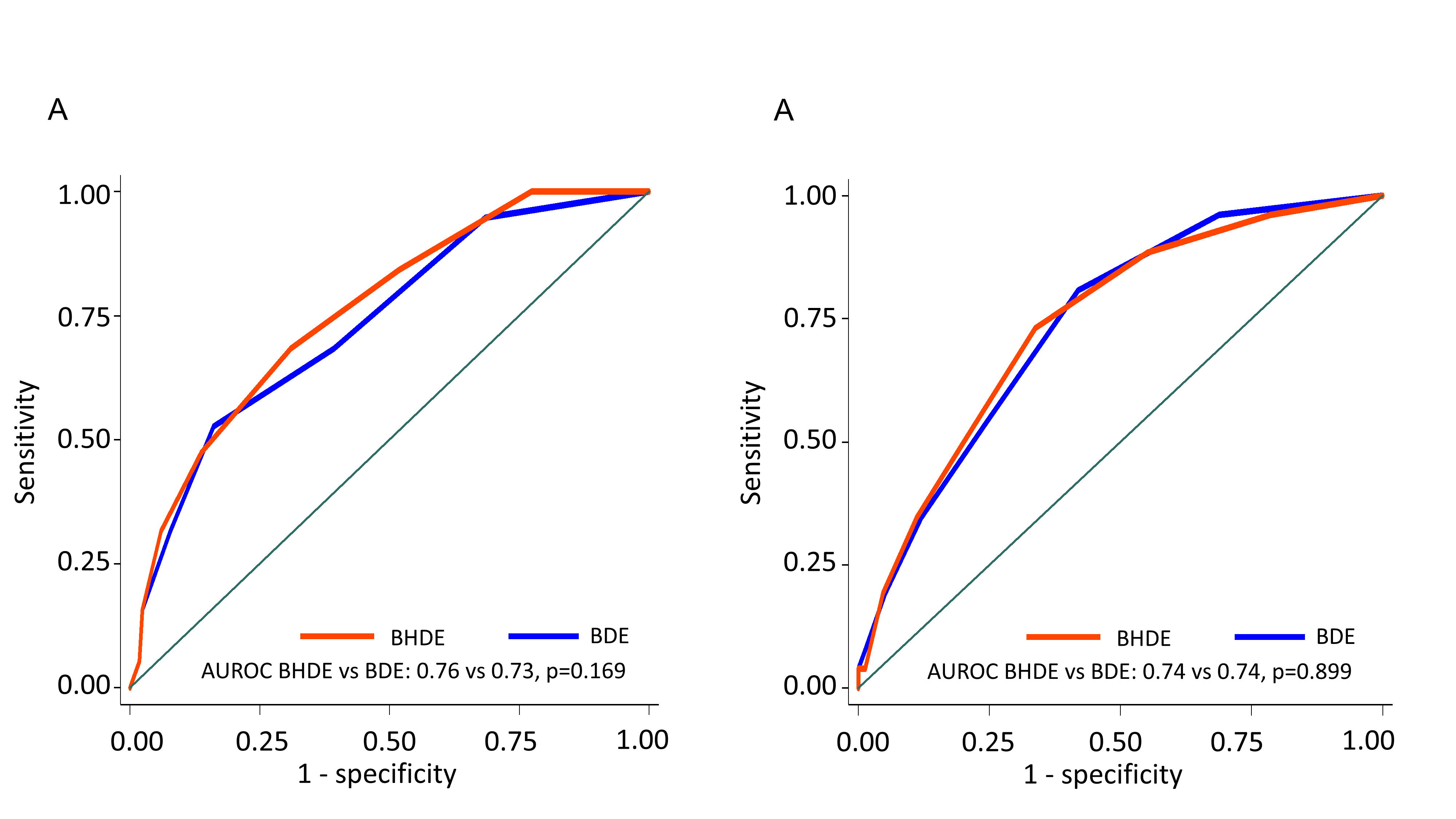

Supplement: Supplementary file 6 — Supplementary Material 6 [file 12890_2023_2557_MOESM6_ESM.tif]

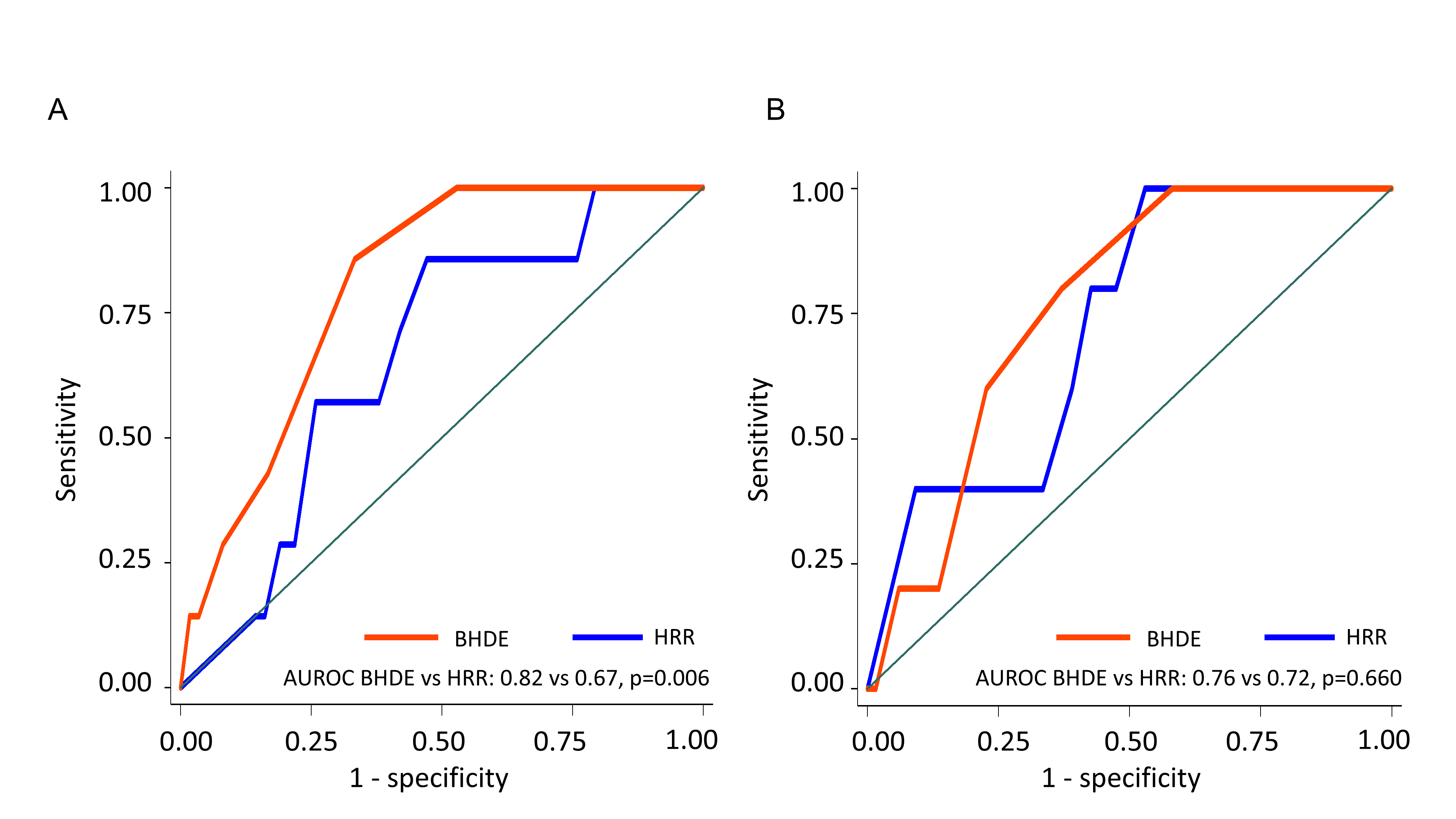

Supplement: Supplementary file 7 — Supplementary Material 7 [file 12890_2023_2557_MOESM7_ESM.tif]

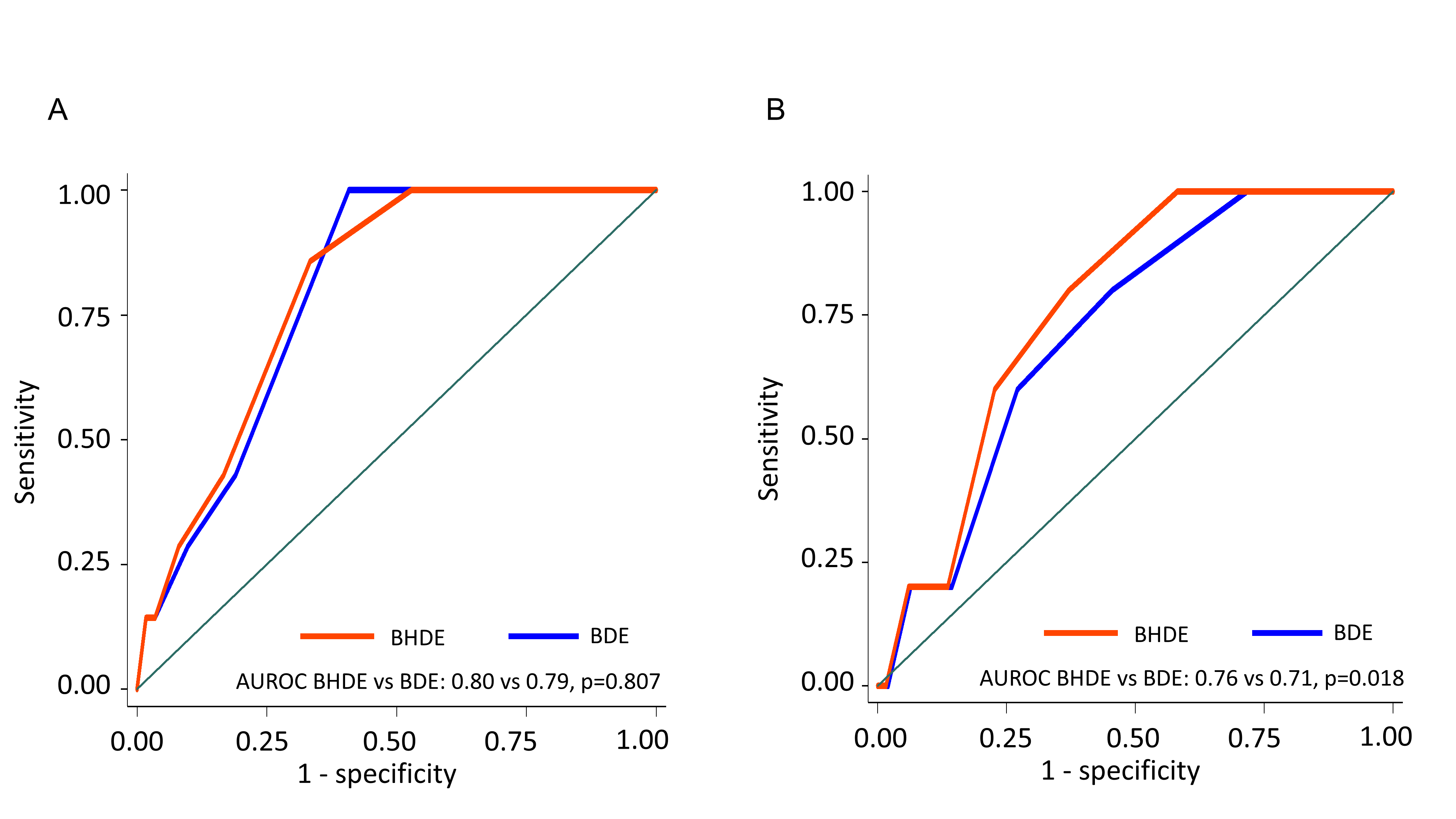

Supplement: Supplementary file 8 — Supplementary Material 8 [file 12890_2023_2557_MOESM8_ESM.tif]
