# Supplementary material for: Prognostic value of the post-exercise heart rate recovery and BHDE-index in chronic obstructive pulmonary disease
Source: BMC Pulm Med. 2023 Jul 17;23:263. doi: 10.1186/s12890-023-02557-7 (PMC10353238; doi:10.1186/s12890-023-02557-7)
Supplement: Supplementary file 9 — Supplementary Material 9 [file 12890_2023_2557_MOESM9_ESM.docx]

**Supplementary Figure legends**

**supplementary figure S1** Receiver operating characteristic curves of post 6-min walk test HRR to the possibility of severe acute exacerbation in derivation cohort (Panel A) and validation cohort (Panel B). HRR, post 6-minute walk test heart rate recovery; ROC, receiver operating characteristic curve

**supplementary figure S2** Kaplan-Meier time-to-event plot and log-rank test for time to first acute severe exacerbation in one year between abnormal HRR and HRR in the derivation cohort (Panel A) and validation cohort (Panel B). B, body mass index; O, airflow obstruction; H, post 6-minute walk test HRR; D, dyspnea score; E, exercise intolerance; HRR, heart rate recovery, CI, confidence interval

**supplementary figure S3** Scatter plots and correlation coefficients between HRR and CAT, mMRC, resting Borg score, post-exercise Borg score and 6MWD in the derivation cohorts (Panel A) and validation cohort (Panel B). CAT: COPD assessment test; mMRC: modified Medical Research Council dyspnea scale; 6MWD: 6-minute walking distance

**supplementary figure S4** Scoring profile of BODE and BHDE index. B, body mass index; O, airflow obstruction; H, post 6-minute walk test HRR; D, dyspnea score; E, exercise intolerance; FEV1, forced expiratory volume in 1 s; HRR, heart rate recovery

**supplementary figure S5**

Receiver operating characteristic curves comparing BHDE with HRR in the derivation cohort (A), and validation cohort (B) in the prediction of occurrence of severe acute exacerbation. B, body mass index; H/HRR, post 6-minute walk test 1-min heart rate recovery; D, dyspnea score; E, exercise intolerance; AUROC, area under the receiver operating characteristic curve

**supplementary figure S6**

Receiver operating characteristic curves comparing BHDE with BDE in the derivation cohort (A), and validation cohort (B) in the prediction of occurrence of severe acute exacerbation. B, body mass index; H/HRR, post 6-minute walk test 1-min heart rate recovery; D, dyspnea score; E, exercise intolerance; AUROC, area under the receiver operating characteristic curve

**supplementary figure S7**

Receiver operating characteristic curves comparing BHDE with HRR in the derivation cohort (A), and validation cohort (B) in the prediction of occurrence of 1-year mortality. B, body mass index; H/HRR, post 6-minute walk test 1-min heart rate recovery; D, dyspnea score; E, exercise intolerance; AUROC, area under the receiver operating characteristic curve

**supplementary figure S8**

Receiver operating characteristic curves comparing BHDE with BDE in the derivation cohort (A), and validation cohort (B) in the prediction of occurrence of 1-year mortality. B, body mass index; H/HRR, post 6-minute walk test 1-min heart rate recovery; D, dyspnea score; E, exercise intolerance; AUROC, area under the receiver operating characteristic curve
