# Supplementary material for: Prognostic value of the post-exercise heart rate recovery and BHDE-index in chronic obstructive pulmonary disease
Source: BMC Pulm Med. 2023 Jul 17;23:263. doi: 10.1186/s12890-023-02557-7 (PMC10353238; doi:10.1186/s12890-023-02557-7)
Supplement: Supplementary file 10 — Supplementary Material 10 [file 12890_2023_2557_MOESM10_ESM.docx]

**Prognostic value of the post-exercise heart rate recovery and BHDE-index in chronic obstructive pulmonary disease**

**Supplementary table S1:** Univariate and multivariate analyses of factors associated with severe acute exacerbation of COPD within one year in the validation cohort.

|  | Univariable analysis | | |  | Multivariable analysis | | |
| --- | --- | --- | --- | --- | --- | --- | --- |
|  | HR | 95% CI | p-value |  | HR | 95% CI | p-value |
| BHDE index | 1.52 | 1.26-1.85 | <0.05 |  | 1.38 | 1.10-1.73 | <0.05 |
| Age, year | 1.02 | 0.98-1.07 | 0.286 |  | - | - | - |
| Airway obstruction, FEV1 (%) | 0.96 | 0.95-0.98 | <0.05 |  | 0.98 | 0.96-0.99 | <0.05 |
| Inhaled corticosteroid | 1.62 | 0.75-3.50 | 0.220 |  | - | - | - |
| Active smoking vs. quitted | 0.29 | 0.04-2.16 | 0.140 |  | - | - | - |
| Severe AE in previous 1 year | 1.43 | 1.09-1.90 | <0.05 |  | 1.20 | 0.90-1.60 | 0.232 |

HR, hazard ratio; CI, confidence interval; BHDE: body mass index (B), post 6-minute walk test 1-min heart rate recovery (H), dyspnea score (D), exercise intolerance (E); FEV1, forced expiratory volume in 1 second; AE: acute exacerbation

**Supplementary table S2:** Univariate and multivariate analyses of factors associated with 1 year mortality of COPD in the derivation cohort.

|  | Univariable analysis | | |  | Multivariable analysis | | |
| --- | --- | --- | --- | --- | --- | --- | --- |
|  | HR | 95% CI | p-value |  | HR | 95% CI | p-value |
| BHDE index | 1.52 | 1.26-1.85 | <0.05 |  | 1.49 | 0.92-2.42 | 0.104 |
| Age, year | 1.20 | 1.06-1.35 | <0.05 |  | 1.21 | 1.05-1.39 | <0.05 |
| Airway obstruction, FEV1 (%) | 0.96 | 0.93-1.00 | 0.055 |  | - | - | - |
| Inhaled corticosteroid | 3.37 | 0.65-17.4 | 0.146 |  | - | - | - |
| Active smoking vs. quitted | 2.77 | 0.62-12.4 | 0.182 |  | - | - | - |
| Severe AE in previous 1 year | 1.43 | 1.09-1.90 | <0.05 |  | 8.18 | 1.75-38.2 | <0.05 |

HR, hazard ratio; CI, confidence interval; BHDE: body mass index (B), post 6-minute walk test 1-min heart rate recovery (H), dyspnea score (D), exercise intolerance (E); FEV1, forced expiratory volume in 1 second; AE: acute exacerbation

**Supplementary table S3:** Univariate and multivariate analyses of factors associated with 1 year mortality of COPD in the validation cohort.

|  | Univariable analysis | | |  | Multivariable analysis | | |
| --- | --- | --- | --- | --- | --- | --- | --- |
|  | HR | 95% CI | p-value |  | HR | 95% CI | p-value |
| BHDE index | 1.49 | 1.02-2.17 | <0.05 |  | 1.41 | 0.94-2.12 | 0.098 |
| Age, year | 1.06 | 0.97-1.16 | 0.199 |  | 1.04 | 0.95-1.13 | 0.453^*^ |
| Airway obstruction, FEV1 (%) | 0.98 | 0.95-1.01 | 0.296 |  | - | - | - |
| Inhaled corticosteroid | 3.99 | 0.70-22.7 | 0.118 |  | - | - | - |
| Active smoking vs. quitted | 5.13 | 0.86-30.7 | 0.073 |  | - | - | - |
| Severe AE in previous 1 year | 2.63 | 0.29-23.5 | 0.387 |  | 1.35 | 0.14-13.1 | 0.796^*^ |

HR, hazard ratio; CI, confidence interval; BHDE: body mass index (B), post 6-minute walk test 1-min heart rate recovery (H), dyspnea score (D), exercise intolerance (E); FEV1, forced expiratory volume in 1 second; AE: acute exacerbation; ^*^Because age and severe AE in previous one year are significant risk factors in derivation cohort, it was put in multivariate analysis for further analysis.
